# Visualizing Aortic Inflammation by Diffusion-Weighted Whole-Body Imaging with Background Body Signal Suppression (DWIBS)

**DOI:** 10.3390/diagnostics15091151

**Published:** 2025-04-30

**Authors:** Asuka Suzuki, Koji Hayashi, Mamiko Sato, Yuka Nakaya, Toyoaki Miura, Naoko Takaku, Toshiko Iwasaki, Yasutaka Kobayashi

**Affiliations:** 1 Department of Rehabilitation Medicine, Fukui General Hospital, 55-16-1 Egami-cho, Fukui 910-8561, Japansatomoko@f-gh.jp (M.S.); tnaoko18@u-fukui.ac.jp (N.T.); 2 Graduate School of Health Science, Fukui Health Science University, 55-13-1 Egami, Fukui 910-3190, Japan; yasutaka_k@fukui-hsu.ac.jp; 3 Department of Neurology, University of Fukui Hospital, 23-3 Matsuoka Shimoaizuki, Eiheiji-cho, Yoshida-gun, Fukui 910-1193, Japan; 4 Department of Radiology, Fukui General Hospital, 55-16-1 Egami-cho, Fukui 910-8561, Japan

**Keywords:** MRI, diagnosis, aortitis, aortic stent, inflammation, ulcer, stent ulcers

## Abstract

A 75-year-old man, with a history of descending thoracic aortic rupture and dissection treated with aortic stenting at 73 years old, was admitted for rehabilitation following recurrent cerebral ischemic attacks. Upon admission, blood tests revealed elevated inflammatory markers, including a C-reactive protein (CRP) level of 10.75 mg/dL and a D-dimer level of 4.2 µg/mL, alongside microcytic anemia. Despite thorough evaluations using computed tomography (CT) and ultrasound, the origin of these abnormalities remained unidentified. Two months later, MRI using diffusion-weighted whole-body imaging with background body signal suppression (DWIBS) revealed hyperintensities in the thoracic aorta. He remained asymptomatic and progressed well during rehabilitation, prompting continued observation. However, three months after admission, he developed hemoptysis. Contrast-enhanced CT showed pneumonia, as well as enhanced lesions in the aortic wall, confirming aortic inflammation. Due to concerns about aortic stent ulceration, an emergency stent graft insertion extending to the superior mesenteric artery was performed. He recovered uneventfully and was discharged. DWIBS is an MRI-based tool that avoids exposure to radiation or contrast agents and is cost-effective. MRI using DWIBS demonstrated high signal accumulations in the aortic wall, indicative of inflammation. These findings suggest that DWIBS holds significant potential as a powerful imaging tool for detecting and assessing inflammation, particularly in the aorta.

**Figure 1:**
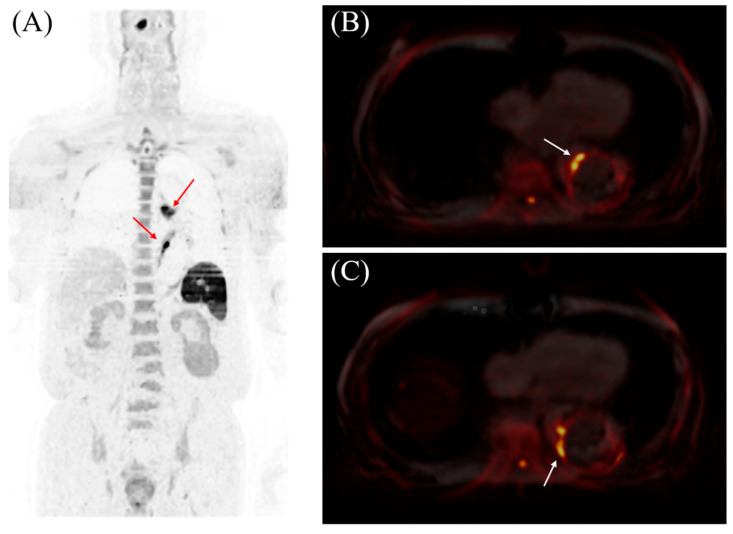
The results of magnetic resonance imaging using diffusion-weighted whole-body imaging with background body signal suppression (DWIBS). (**A**) Three-dimensional rendering of the DWIBS data demonstrates high signal intensity surrounding the aorta (red arrows). (**B**,**C**) Axial slices from the DWIBS dataset (original images) show hyperintensity within the aortic wall (white arrows). DWIBS, pioneered by Takahara et al. in 2004, is an advanced MRI-based technique for comprehensive whole-body cancer screening [1]. This technique employs a short τ inversion recovery echo-planar imaging sequence with free breathing for the highly sensitive detection of diffusion-restricted lesions, such as cancers and abscesses, by leveraging the movement of water molecules to create sharp contrasts between abnormal and normal structures across the entire body simultaneously [1,2,3,4]. This provides a level of convenience comparable to positron emission tomography–computed tomography (PET-CT) [3]. DWIBS offers both cost-effectiveness and the advantage of being non-invasive, as it eliminates the need for expensive radionuclides that may otherwise pose a risk of radiation exposure [3]. Additionally, DWIBS has been reported to detect inflammatory pathology, with its most common application being Takayasu aortitis [5,6]. In addition, there is a case of aortic inflammation related to giant cell arteritis detected by DWIBS [7]. In this case, high DWI signals were observed along the aortic wall, indicating such inflammation. Cardiovascular surgeons suspected that the inflammation was associated with an aortic stent ulcer, but conclusive evidence to support this was unavailable. Nevertheless, based on the previous reports and our case, we believe that DWIBS holds promise as a useful modality for detecting inflammatory lesions, particularly in the aorta.

**Figure 2:**
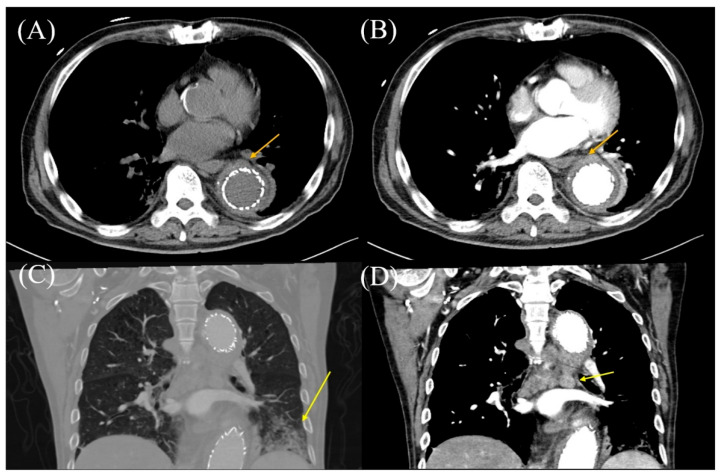
The results of computed tomography (CT) with or without contrast agent. (**A**) Thoracic CT without a contrast agent reveals that the aortic wall has Hounsfield units (HU) of 21 (arrow). (**B**) Thoracic CT with a contrast agent reveals areas of enhancement in the aortic wall, located outside the aortic stent, with an HU of 82 (arrow). (**C**) Thoracic CT in the coronal section reveals pneumonia (arrow). (**D**) Contrast-enhanced thoracic CT reveals mediastinal lymph node enlargement (arrow).

## Data Availability

The data presented in this study are available on request from the corresponding author. Due to patient privacy and ethical considerations, the data are not publicly accessible.
